# Correction: Synthesis of γ-alumina (Al_2_O_3_) nanoparticles and their potential for use as an adsorbent in the removal of methylene blue dye from industrial wastewater

**DOI:** 10.1039/c8na90001a

**Published:** 2018-10-08

**Authors:** Shafqat Ali, Yasir Abbas, Zareen Zuhra, Ian S. Butler

**Affiliations:** The Key Laboratory of Advanced Materials of Ministry of Education, School of Material Science and Engineering, Tsinghua University Beijing 100084 China shafqatali@mail.tsinghua.edu.cn +86-10-64421693; State Key Laboratory of Chemical Resource Engineering, Institute of Science, Beijing University of Chemical Technology Beijing 100029 P. R. China; Department of Chemistry, McGill University Montreal QC H3A 2K6 Canada

## Abstract

Correction for ‘Synthesis of γ-alumina (Al_2_O_3_) nanoparticles and their potential for use as an adsorbent in the removal of methylene blue dye from industrial wastewater’ by Shafqat Ali *et al.*, *Nanoscale Adv.*, 2018, DOI: 10.1039/c8na00014j.

The authors wish to amend the sections of the manuscript detailed below. These corrections are due to inaccuracies in the adsorption activity, as well as in the surface area of the adsorbent.

The corrections are as follows:

(1) On page 1, the sentence including “4.13 nm and the surface area is 112.9 m^2^ g^−1^” should instead read as “4.55 nm and the surface area is 120.7 m^2^ g^−1^”.

(2) On page 1, in the sentence including “490 to 2210 mg g^−1^” should instead read as “483 to 2190 mg g^−1^”.

(3) On page 3 (left column), the sentence including “16.00 m^2^ g^−1^” should instead read as “120.7 m^2^ g^−1^”, and “4.13 nm” should instead read as “4.55 nm”.

(4) On page 4 (left column), the sentence including “808 mg g^−1^” should instead read as “790 mg g^−1^”.

(5) Page 5 (right column), the sentence including “868 to 1000 mg g^−1^” should instead read as “849 to 1000 mg g^−1^”.

(6) Page 5 (right column), the sentence including “422, 431, 481 and 490 mg g^−1^” should instead read as “417, 425, 476 and 483 mg g^−1^”.

(7) Page 5 (right column), the sentence including “1812, 2061, 2164 and 2210 mg g^−1^” should instead read as “1800, 2043, 2110 and 2190 mg g^−1^”.

(8) Page 5 (left column), the sentence including “868 to 1000 mg g^−1^” should instead read as “849 to 1000 mg g^−1^”.

**Fig. 1:**
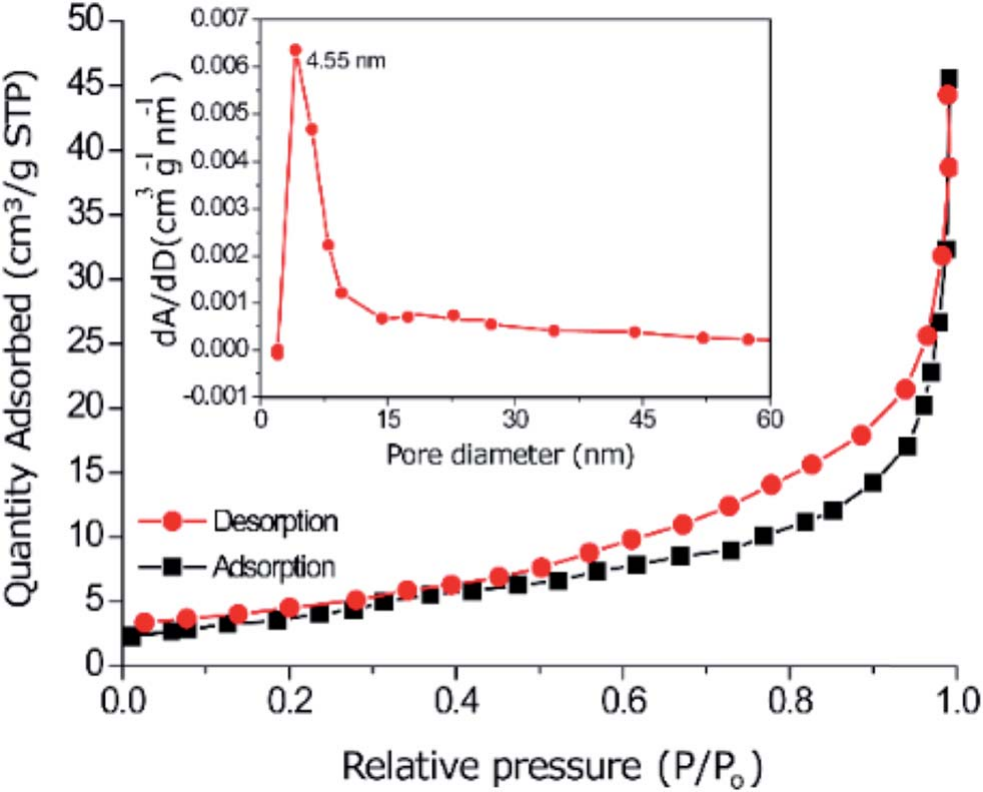
Nitrogen adsorption–desorption isotherms and pore size distribution of the nanoparticles.

**Fig. 2:**
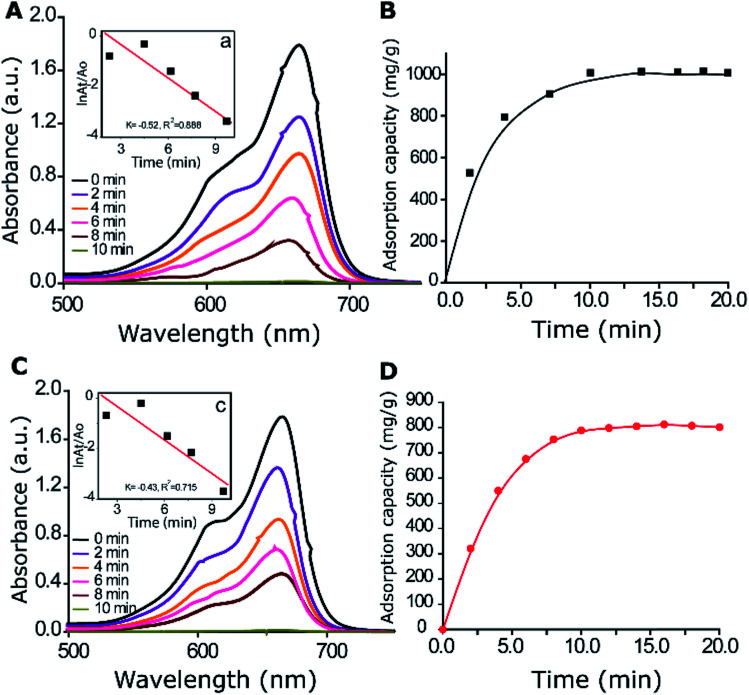
Effect of contact time of 100 ppm of MB on 10 mg of the γ-alumina nanoparticles at pH 9.0 (A) and pH 7.0 (C), and their adsorption capacities (B) and (D), respectively. The inset is the linear correlation between ln[*A*_*t*_/*A*_0_] and time for the degradation of MB.

**Fig. 3:**
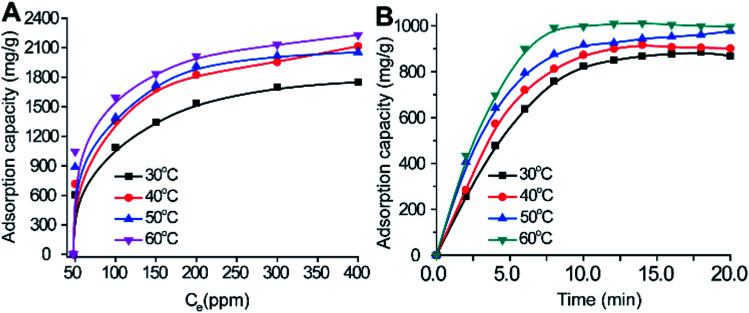
Adsorption capacities at certain temperatures (30, 40, 50 and 60 °C): 100 ppm (A), and 50, 100, 150, 200, 300, and 400 ppm (B) of MB on 10 mg of the γ-alumina nanoparticles within 10 min at pH 9.0.

**Fig. 4:**
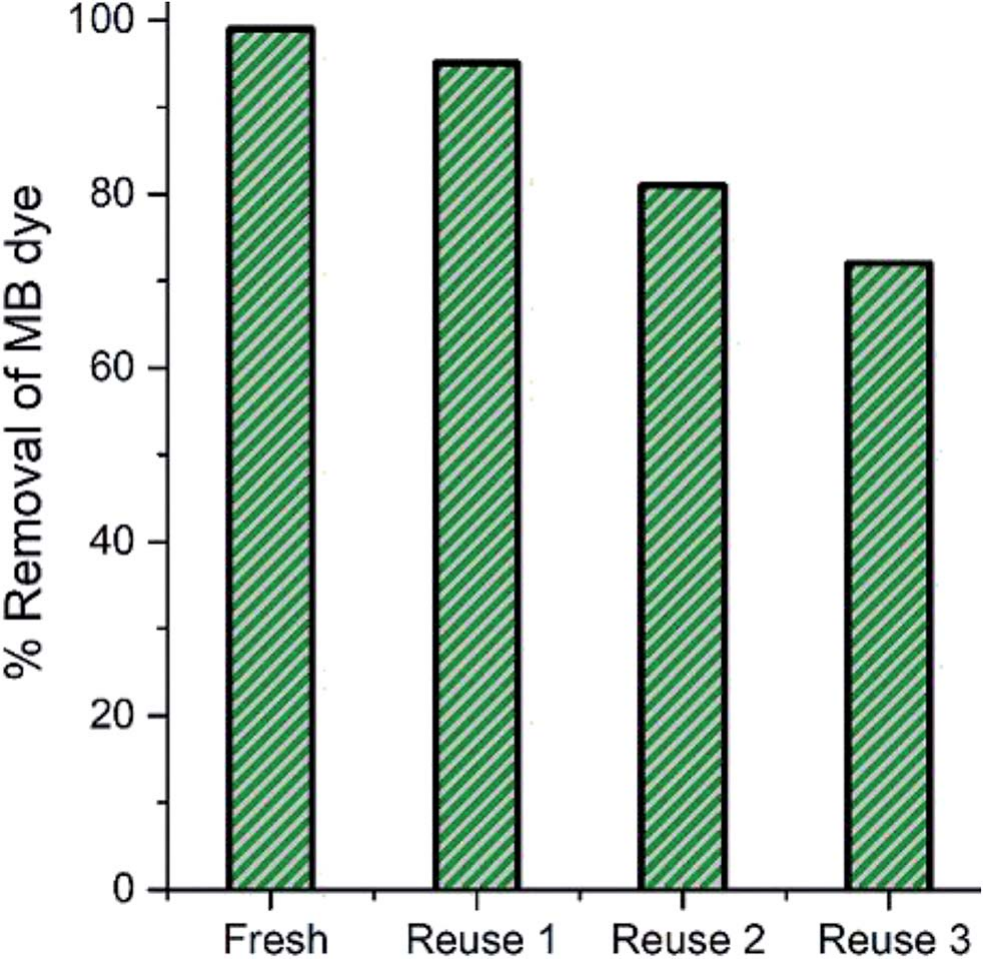
Catalytic activity of MB in the presence of recycled nanoparticles under the same conditions.

The Royal Society of Chemistry apologises for these errors and any consequent inconvenience to authors and readers.

## Supplementary Material

